# Lipoprotein(a) and Pulmonary Embolism Severity-A Retrospective Data Analysis

**DOI:** 10.3389/fcvm.2022.808605

**Published:** 2022-02-07

**Authors:** Paul Gressenberger, Florian Posch, Moritz Pechtold, Katharina Gütl, Viktoria Muster, Philipp Jud, Jakob Riedl, Günther Silbernagel, Ewald Kolesnik, Johannes Schmid, Reinhard B. Raggam, Marianne Brodmann, Thomas Gary

**Affiliations:** ^1^Division of Angiology, Department of Internal Medicine, Medical University of Graz, Graz, Austria; ^2^Division of Oncology, Department of Internal Medicine, Medical University of Graz, Graz, Austria; ^3^Division of Cardiology, Department of Internal Medicine, Medical University of Graz, Graz, Austria; ^4^Division of General Radiology, Department of Radiology, Medical University of Graz, Graz, Austria

**Keywords:** Lipoprotein(a), pulmonary embolism, severity, venous thromboembolism, Lp(a)

## Abstract

**Aim:**

We aimed to investigate a correlation between PE severity and Lp(a) levels.

**Methods:**

We performed a retrospective data analysis from our medical records of PE patients admitted to the University Hospital Graz, Austria. Patients with an Lp(a) reading within a 1-year interval before and after PE diagnosis were included. In accordance with the 2019 ESC guidelines for the diagnosis and management of acute PE, severity assessment was carried out classifying patients into four groups: low risk (LR), intermediate low risk (IML), intermediate high risk (IMH) and high risk (HR). The study period of interest was between January 1, 2002 and August 1, 2020.

**Results:**

We analyzed 811 patients with PE, of whom 323 (40%) had low-risk PE, 343 (42%) had intermediate-low-risk PE, 64 (8%) had intermediate-high-risk PE, and 81 (10%) had high-risk PE, respectively. We did not observe an association between PE severity and Lp(a) concentrations. In detail, median Lp(a) concentrations were 17 mg/dL [25–75th percentile: 10-37] in low-risk PE patients, 16 mg/dL [10–37] in intermediate-low-risk PE patients, 15mg/dL [10–48] in intermediate-high-risk PE patients, and 13mg/dL [10–27] in high-risk PE patients, respectively (Kruskal-Wallis *p* = 0.658, p for linear trend = 0.358).

**Conclusion:**

The current findings suggest no correlation between PE severity and Lp(a) levels.

## Highlights

- We aimed to investigate a correlation between PE severity and Lp(a) levels.
- Potential pathomechanisms of Lp(a) include similarities of Lp(a) to plasminogen, resulting in a decrease of plasmin synthesis and inhibition of fibrinolysis, which is mainly observed under laboratory conditions. It, however, remains elusive whether this inhibitory effect is strong enough to play a significant role in the development of venous thrombotic events (VTE) such as pulmonary embolism (PE).
- The current findings suggest no correlation between PE severity and Lp(a) levels.

## Introduction

Lipoprotein(a) (Lp(a)) is a genetically determined low-density lipoprotein (LDL) particle. In the absence of acute inflammation, the Lp(a) level is stable through an individual's lifetime, regardless of lifestyle (1). Elevated Lp(a) levels are strongly associated with the development of atherosclerotic cardiovascular diseases (ASCVD) such as stroke, peripheral artery disease or coronary heart disease (2, 3). A Lp(a) level over 50 mg/dL is generally considered as an additional factor that indicates a high risk of ASCVD, whereas the highest risk is strongly restricted to those with very high Lp(a)-concentrations (3). Therefore, the European Society of Cardiology (ESC) recommends measuring Lp(a) levels in selected patients at high risk of ASCVD (4). Potential pathogenic mechanisms of Lp(a) include their propensity to oxidize after entry into the vessel wall, creating highly immunogenic and proinflammatory phospholipids, the presence of lysine binding sites that allow accumulation in the arterial wall, and similarities of Lp(a) to plasminogen, resulting in a decrease of plasmin synthesis and inhibition of fibrinolysis (5, 6). It, however, remains elusive whether this inhibitory effect is strong enough to play a significant role in the development of venous thrombotic events (VTE) such as pulmonary embolism (PE) (7).

PE is a leading cause of death worldwide, especially when massive PE is present (8, 9). In the current ESC guidelines for the management of PE (10), PE-related severity is stratified based on clinical presentation and factors contributing to haemodynamic collapse, reflecting acute right ventricular (RV) dysfunction (11). According to these guidelines we aimed to investigate a potential correlation between PE severity and Lp(a) levels in a single-center cohort by retrospective data analysis.

## Methods

### Study Design and Patient Population

We performed a retrospective chart review study from our medical records of PE patients with an available Lp(a) value admitted to the University Hospital Graz, Austria. At our center, admission of patients with newly-diagnosed PE to an inpatient ward is local standard-of-care. Although Lp(a) is thought to be relatively stable within patients over time, latency between PE diagnosis and Lp(a) determination was a maximum of 1 year, i.e., only patients with an Lp(a) reading within a 1-year interval before and after PE diagnosis were included. In accordance with the 2019 ESC guidelines (10) for the diagnosis and management of acute PE, severity assessment was carried out classifying patients into four groups: low risk (LR), intermediate low risk (IML), intermediate high risk (IMH) and high risk (HR). These guidelines report a PE risk stratification based on immediate and early mortality risk. The presence of haemodynamic instability is the main determinant to classify patients as having a high risk PE. In these guidelines other patients are divided into intermediate-high risk (no hemodynamic instability, but clinical criteria of severity, positive Pulmonary Embolism Severity Index (PESI) simplified positive Pulmonary Embolism Severity Index (sPESI), and both signs of right ventricular (RV) dilation and positive troponin), intermediate-low risk (no haemodynamic instability, presence of clinical criteria of severity, positive PESI or sPESI and either RV dilation or positive troponin), or low risk (no hemodynamic instability and a negative PESI or sPESI) (10, 11). According to these guidelines, for patients with no hemodynamic instability, signs of RV dilation and positive troponin were included in the risk stratification as well as Pulmonary Embolism Severity Index (PESI) score was assigned based on the variables of age, sex, previous PE, cancer, comorbidities, O_2_-saturation, systolic blood pressure and heart rate. RV dysfunction was assessed by computed tomography (CT) by specialized radiologists, and in selected cases by point-of-care echocardiography. CT criteria for RV dysfunction included a ratio of right to left ventricular diameter (RV/LV) > 1, bulging of the interventricular septum and reflux of contrast media into the inferior vena cava and hepatic veins. Echocardiographic assessments of RV dysfunction were performed on a case-by-case basis by treating physicians at our acute care treatment facilities. Laboratory data (estimated glomerular filtration rate (eGFR), Troponin T, (NT-pro) Brain Natriuretic Peptide) were extracted as close as possible to Lp(a) assessment date. In contrast, comorbidities (cancer, COPD, asthma, heart failure, kidney disease) were extracted within a time frame of seven days prior and after PE diagnosis date. The study period of interest was between January 1, 2002 and August 1, 2020. The study protocol was approved by the Ethics Committee (EK 32-646 ex 19/20) of the Medical University of Graz.

### Statistical Analysis

All statistical analyses were performed with Stata (Windows Version 17.0, Stata Corp., Houston, TX, USA). Continuous variables were summarized as medians [25–75^th^ percentile], and count data as absolute frequencies (%). Correlations between two continuous variables were evaluated with Spearman's rank-based correlation coefficient. The primary aim was the association between PE severity as indicated by the ESC PE risk stratification (4-level ordinal variable defined above) and the Lp(a) levels both as a continuous variable and as a binary variable dichotomized at a pre-defined cut-off at 50 mg/dL. For these analyses, we employed Kruskal-Wallis tests, simple and multiple linear regression models (multiple linear regression adjusted for age and sex), F-tests for linear trend, box plots, χ^2^-tests, and Fisher's exact tests, as appropriate. In a pre-specified sensitivity analysis, we examined whether extremely high levels of Lp(a), defined by three Lp(a) cut-offs >80 mg/dL, >120 mg/dL, and >160 mg/dL, were associated with high-risk PE.

## Results

### Cohort Description

We analyzed 811 patients with PE, of whom 323 (40%) had low-risk PE, 343 (42%) had intermediate-low-risk PE, 64 (8%) had intermediate-high-risk PE, and 81 (10%) had high-risk PE, respectively (Table 1). Median Lp(a) concentration was 15 mg/dL [25–75th percentile: 10-35, range: 0.6 – 254]. Median time between Lp(a) measurement and index PE was 0 days [25–75th percentile:−6–1 days, range:−359–361 days]. Higher Lp(a) did not correlate with age (Spearman's ρ = 0.04, *p* = 0.271), and was comparable between males and females (median Lp(a). 15 vs. 16, *p* = 0.181). Neither BMI, nor eGFR, nor Troponin T, nor BNP, nor comorbidities at PE diagnosis, including cancer, asthma, COPD, heart failure, and kidney disease, were associated with Lp(a) levels.

**Table 1:** Baseline characteristics of the study population (*n* = 811).

**Variables**	**Overall (*n* = 811)**	**Lp(a) ≤50 mg/dL (*n* = 681)**	**Lp(a) > 50 mg/dL (*n* = 130)**	***p*-value**
Age at PE diagnosis (years)	69 [54–80]	69 [53–80]	69 [56–79]	0.792
Female sex	595 (51%)	418 (49%)	177 (55%)	0.106
BMI (kg/m^2^)^*^	27 [24–30]	27 [24–30]	26 [24–30]	0.730
eGFR (ml/min/1.73 m^2^)^*^	69 [52–85]	68 [52–85]	72 [50–86]	0.563
Cancer at PE diagnosis^**^	73 (9%)	59 (9%)	14 (11%)	0.442
Asthma at PE diagnosis^**^	13 (2%)	11 (2%)	2 (2%)	0.999
COPD at PE diagnosis^**^	68 (8%)	54 (8%)	14 (11%)	0.284
Heart failure at PE diagnosis^**^	61 (8%)	46 (7%)	15 (12%)	0.058
Kidney disease at PE diagnosis^**^	114 (14%)	94 (14%)	20 (16%)	0.635
Troponin T (pg/mL)^*^	10 [10–12]	10 [10–11]	10 [10–19]	0.417
Brain natriuretic peptide (pg/mL)^*^	600 [125–2518]	586 [117–2518]	662 [172–2484]	0.486
PE risk stratification	/	/	/	0.430
Low-risk	323 (40%)	268 (39%)	55 (42%)	/
Intermediate-Low-risk	343 (42%)	293 (43%)	50 (38%)	/
Intermediate-High-risk	64 (8%)	50 (7%)	14 (11%)	/
High-risk	81 (10%)	70 (10%)	11 (8%)	/

*Distribution overall and by Lipoprotein(a) status. We used a Lp(a) cut-off at 50 mg/dL. Reported data are medians [25–75th percentile] for continuous variables, and absolute frequencies (column %) for count data. P-values are from rank-sum tests and χ^2^-tests, as appropriate. Lp(a), Lipoprotein(a); PE, Pulmonary embolism*.

* *closest reading to Lp(a) assessment date*.

** *reported within 7 days prior and after PE diagnosis. Troponin T and BNP were not included in this model, as cumulative missingness in these two variables would have led to the final regression model being fitted in only n = 370 patients*.

### Lp(a) Concentration by PE Severity

**Figure 1:**
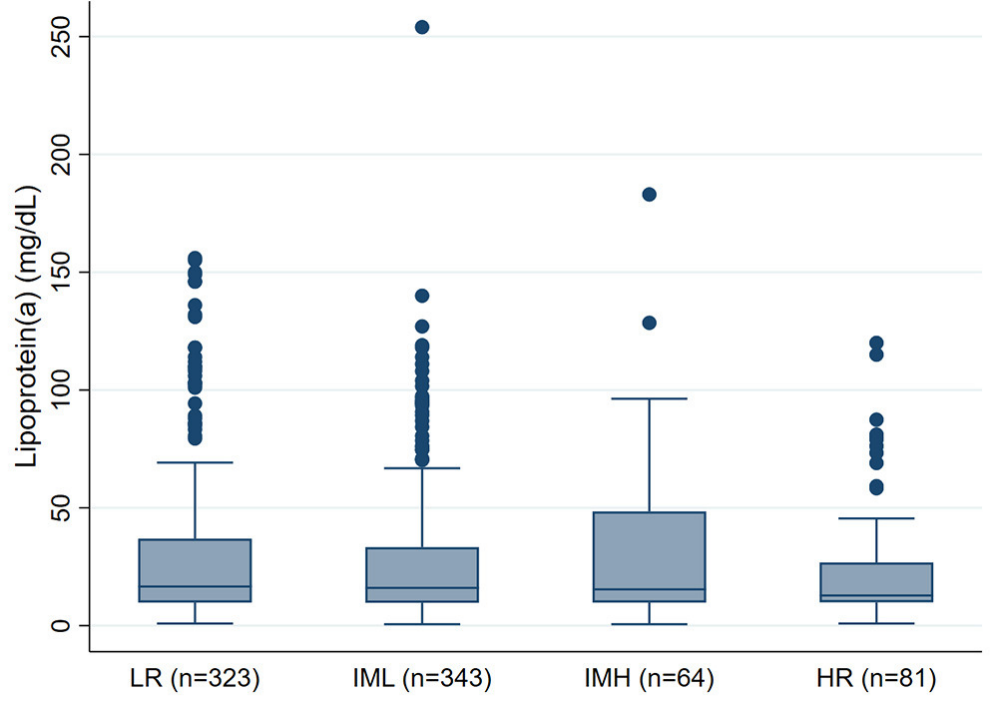
Boxplots of Lipoprotein(a) levels according to PE severity (*n* = 811). PE, Pulmonary embolism; LR, Low-risk PE; IML, Intermediate-Low-risk PE; IMH, Intermediate-High-risk PE; HR, High-risk PE.

We did not observe an association between PE severity and Lp(a) concentrations. In detail, median Lp(a) concentrations were 17mg/dL [25–75th percentile: 10-37] in low-risk PE patients, 16mg/dL [10–33] in intermediate-low-risk PE patients, 15 mg/dL [10–48] in intermediate-high-risk PE patients, and 13mg/dL [10–27] in high-risk PE patients, respectively (Kruskal-Wallis *p* = 0.658, p for linear trend = 0.358, Figure 1). This result prevailed also after multivariable adjustment for age, sex, BMI, eGFR, Troponin T, BNP, and comorbidities including cancer, asthma, COPD, and heart failure (Adjusted p for association between Lp(a) and PE severity = 0.212, Table 2). In this multivariable model, heart failure emerged as the only statistically significant predictor of Lp(a) levels.

**Table 2:** A multiple linear regression model of Lipoprotein(a).

**Variable**	**β coefficient**	**95%CI**	***p*-value**
Age at PE diagnosis (per 5 years increase)	0.99	−0.03–2.01	0.057
Female sex	2.78	−2.63–8.19	0.314
PE risk stratification	/	/	0.212
Low-risk	Ref.	Ref.	Ref.
Intermediate-Low-risk	−6.53	−13.04-(-0.02)	0.049
Intermediate-High-risk	−4.87	−15.67–5.94	0.377
High-risk	−7.03	−16.66–2.61	0.153
BMI (per 5 kg/m^2^ increase)	0.39	−0.97–1.76	0.572
eGFR (per 5 ml/min/1.73 m^2^ increase)	0.36	−0.26–0.98	0.260
Cancer at PE diagnosis	1.57	−8.05–11.19	0.749
Asthma and/or COPD at PE diagnosis	4.82	−4.28–13.92	0.298
Heart failure at PE diagnosis	13.18	1.96–24.39	0.021
Constant	10.31	−10.31–30.93	0.327

*The β coefficient represents the change in Lp(a) per one unit change in the respective variable. 95%CI, 95% confidence interval; p, Wald test p-value; PE, Pulmonary embolism; Ref., Reference category*.

### Sensitivity Analysis–Very High Levels of Lp(a)

In this sensitivity analysis, we examined whether extremely high levels of Lp(a), defined by three Lp(a) cut-offs >80 mg/dL, >120 mg/dL, and >160 mg/dL, are associated with high-risk PE, which was not the case (Table 3).

**Table 3:** Exploratory analysis of extremely high Lp(a) levels and high-risk PE according to three ascending cut-offs.

**Cut-off**	**Group**	**No high-risk PE (*n* = 730)**	**High-risk PE (*n* = 81)**	***p*-value**
80 mg/dl	Lp(a) ≤ 80 mg/dL (*n* = 748)	672 (92%)	76 (94%)	0.572
	Lp(a) > 80 mg/dL (*n* = 63)	58 (8%)	5 (6%)	
120 mg/dL	Lp(a) ≤ 120 mg/dL (*n* = 797)	716 (98%)	81 (100%)	0.383
	Lp(a) > 120 mg/dL (*n* = 14)	14 (2%)	0 (0%)	
160 mg/dL	Lp(a) ≤ 160 mg/dL (*n* = 809)	728 (99%)	81 (100%)	0.999
	Lp(a) > 160 mg/dL (*n* = 2)	2 (1%)	0 (0%)	

*PE, Pulmonary embolism; p, p-value from χ^2^-tests or Fisher's exact test; as appropriate, Lp(a), Lipoprotein(a)*.

## Discussion

While several studies have shown that elevated Lp(a) levels are a causal risk factor for the development of ASCVD, the role of Lp(a) as a risk factor for VTE remains controversial (5, 7). There is evidence that Lp(a) inhibits fibrinolysis due to the similarity between apolipoprotein(a) and plasminogen (6). These potential mechanisms, however, have explicitly been described in *in-vitro* studies (5, 12). Thus, it remains unknown whether this inhibitory effect plays a relevant role in the global fibrinolytic activity of the circulating blood that depends on many coagulation factors.

Due to impaired fibrinolysis, elevated Lp(a) levels may increase plasma clot density in patients with VTE (6). In this regard, we expected a correlation between PE severity and elevated Lp(a) levels. However, we did not observe any association between PE severity and Lp(a) concentrations. As the highest risk is strongly restricted to those with very high Lp(a)-concentrations, we also performed a sensitivity analysis, where we examined whether extremely high levels of Lp(a), are associated with high-risk PE, which was not the case. Thus, our results suggest that the fibrinolytic effect of Lp(a) may not significantly affect PE severity.

Several studies using different Lp(a) cut-off values aimed to find associations between elevated Lp(a) and VTE [13−15]. Vormittag et al. (13) for example did not find a significant association between Lp (a) plasma levels and the risk of VTE. In contrast other studies such as that from von Depka et al. (14) and that from Marcucci et al. (15) found strong associations between Lp (a) plasma levels and the risk of VTE. The cause of this contrariety is unknown. A recent study tried to clarify the conflicting results and tested whether an inhibitory effect of Lp(a) could only be visible in clot lysis assays with a relatively high tissue plasminogen activator concentration but did not find any correlation between Lp(a) concentration and lysis time (7).

Boffa et al. (12) demonstrated that a potent reduction of Lp(a) in human subjects with high Lp(a) does not affect ex *vivo* clot lysis or biomarkers of coagulation and fibrinolysis. A recent meta-analysis confirmed the questionable role of Lp(a) as a risk factor for VTE (16). Recent data revealed that only Lp(a) concentrations above the 95th percentile may be associated with an increased risk for venous thromboembolism (17). In our study, however, extremely high levels of Lp(a) were also not associated with high-risk PE.

These findings are in line with several other studies showing no association of elevated Lp(a) with deep venous thrombosis (18, 19). One potential reason why Lp(a) primarily promotes ASCVD rather than VTE could be the difference in the etiology of the diseases. VTE represents a different form of thrombosis than ischemic stroke, myocardial infarction or critical limb ischemia where atherosclerosis is concomitantly present. The association of Lp(a) with ASCVD may be attributable through its proatherogenic and proinflammatory components, such as oxidized phospholipids as primary mechanisms (5, 6). In contrast, atherosclerosis does not occur in veins. Pathogenesis of VTE can be explained by using the Virchow's Triad: stasis of blood, hypercoagulability, and endothelial vessel wall injury; which come in to effect after surgery, trauma, immobility or in cancer patients (20). Furthermore, compared with arterial thrombosis, venous thrombosis has a more fibrin-rich and platelet-poor consistency (19).

Last but not least it is noteworthy, that there is rising evidence that statins may be beneficial in preventing VTE (21). Interestingly, a recent meta-analysis revealed that statins can even significantly increase plasma Lp(a) levels (22). This is of some clinical significance, as it underscores the relevance of our findings, that elevated Lp(a) is not linked to VTE. Although the mechanism of action for statins in prevention of VTE is not entirely understood, it could offer new treatment targets.

Our study has several limitations since it is a retrospective data analysis and was based on a single center. First it is quite possible, that in a larger population with more participants with extremely high Lp(a) concentrations, more high risk PE's may have been observed. In addition, although Lp(a) is thought to be relatively stable within patients over time, in our study latency between PE diagnosis and Lp(a) determination was a maximum of 1 year. Thus, it cannot be excluded that Lp(a) measurements directly at the time point of PE diagnosis may have revealed slightly different estimates of the association between Lp(a) and PE severity. Next, as Lp(a) might affect fibrinolytic activity (5, 6), associations of Lp(a) levels and morphological thrombus burden may have been an important research question. However, assessment of thrombus burden in CT scans is challenging. A potential technique would be measurement of thrombus volume by means of thrombus segmentation in CT, which would require very extensive analyses of CT data that was out of the scope and resources of our study. Additionally, clot volume from CT segmentation may not represent overall clot burden, as measurements can be influenced by artifacts, peripheral clots may be underestimated in CT and additional extrapulmonary thrombus material is not represented, which will distort associations. We therefore did not include clot size data in our analysis.

In conclusion we did not observe an association between Lp(a) levels and PE severity. In light of our observations the antifibrinolytic effect of Lp(a) seems to play no significant role in the fibrinolytic activity of the circulating blood in real life, in line with findings of several other studies. Nonetheless our results should encourage other researchers to address potential procoagulant properties of Lp(a) in further studies.

## Data Availability Statement

The raw data supporting the conclusions of this article will be made available by the authors, without undue reservation.

## Ethics Statement

The studies involving human participants were reviewed and approved by the Ethics Committee of the Medical University of Graz. Written informed consent for participation was not required for this study in accordance with the national legislation and the institutional requirements.

## Author Contributions

All authors have contributed significantly to the paper, they understand and endorse it. They have read and approved the version being submitted for publication. The article is original work of the authors.

## Conflict of Interest

The authors declare that the research was conducted in the absence of any commercial or financial relationships that could be construed as a potential conflict of interest.

## Publisher's Note

All claims expressed in this article are solely those of the authors and do not necessarily represent those of their affiliated organizations, or those of the publisher, the editors and the reviewers. Any product that may be evaluated in this article, or claim that may be made by its manufacturer, is not guaranteed or endorsed by the publisher.
